# Characterization of virulence and antimicrobial resistance genes of *Aeromonas media* strain SD/21–15 from marine sediments in comparison with other *Aeromonas* spp.

**DOI:** 10.3389/fmicb.2022.1022639

**Published:** 2022-12-01

**Authors:** Saurabh Dubey, Eirill Ager-Wick, Bo Peng, Øystein Evensen, Henning Sørum, Hetron Mweemba Munang’andu

**Affiliations:** ^1^Section for Experimental Biomedicine, Department of Production Animal Clinical Sciences, Faculty of Veterinary Medicine, Norwegian University of Life Sciences, Ås, Norway; ^2^State Key Laboratory of Biocontrol, Guangdong Key Laboratory of Pharmaceutical Functional Genes, School of Life Sciences, Southern Marine Science and Engineering Guangdong Laboratory (Zhuhai), Sun Yat-sen University, Higher Education Mega Center, Guangzhou, China; ^3^Department of Paraclinical Sciences, Faculty of Veterinary Medicine, Norwegian University of Life Sciences, Ås, Norway; ^4^Faculty of Biosciences and Aquaculture, Nord University, Bodø, Norway

**Keywords:** *Aeromonas media*, antimicrobial resistance, virulence, plasmid, intrinsic–extrinsic, whole genome sequencing

## Abstract

*Aeromonas media* is a Gram-negative bacterium ubiquitously found in aquatic environments. It is a foodborne pathogen associated with diarrhea in humans and skin ulceration in fish. In this study, we used whole genome sequencing to profile all antimicrobial resistance (AMR) and virulence genes found in *A*. *media* strain SD/21–15 isolated from marine sediments in Denmark. To gain a better understanding of virulence and AMR genes found in several *A*. *media* strains, we included 24 whole genomes retrieved from the public databanks whose isolates originate from different host species and environmental samples from Asia, Europe, and North America. We also compared the virulence genes of strain SD/21–15 with *A*. *hydrophila*, *A*. *veronii*, and *A*. *salmonicida* reference strains. We detected *Msh* pili, tap IV pili, and lateral flagella genes responsible for expression of motility and adherence proteins in all isolates. We also found *hylA*, *hylIII*, and *TSH* hemolysin genes in all isolates responsible for virulence in all isolates while the *aerA* gene was not detected in all *A*. *media* isolates but was present in *A*. *hydrophila, A*. *veronii*, and *A*. *salmonicida* reference strains. In addition, we detected *LuxS* and *mshA-Q* responsible for quorum sensing and biofilm formation as well as the ferric uptake regulator (*Fur*), heme and siderophore genes responsible for iron acquisition in all *A*. *media* isolates. As for the secretory systems, we found all genes that form the T2SS in all isolates while only the *vgrG1*, *vrgG3*, *hcp*, and *ats* genes that form parts of the T6SS were detected in some isolates. Presence of *bla*_MOX-9_ and *bla*_OXA-427_ β-lactamases as well as *crp* and *mcr* genes in all isolates is suggestive that these genes were intrinsically encoded in the genomes of all *A*. *media* isolates. Finally, the presence of various transposases, integrases, recombinases, virulence, and AMR genes in the plasmids examined in this study is suggestive that *A*. *media* has the potential to transfer virulence and AMR genes to other bacteria. Overall, we anticipate these data will pave way for further studies on virulence mechanisms and the role of *A*. *media* in the spread of AMR genes.

## Introduction

*Aeromonas media* was first reported as a new species by Allen et al. (1983) who isolated the bacterium from River Avon in Hampshire, England. Since then, it has been reported from sewage, sludge, lakes, rivers, and drinking water (Singh, 2000; Picao et al., 2008; Picão et al., 2008; Figueira et al., 2011; Pablos et al., 2011). In humans, *A*. *media* has mostly been isolated from diarrhea patients (Singh, 2000) while in fish it has been linked to skin ulcerations (Lü et al., 2016). In fish, it has been isolated from Koi carp (Cyprinus carpio; Lü et al., 2016), catfish (*Clarias batrachus*; Singh, 2000), bluntnose bream (*Megalobrama amblycephala*), eel (*Anguilla anguilla*; Yi et al., 2013), southern black bream (*Acanthopagrus butcheri*; Zhou et al., 2013), and crucian carp (*Carassius carassius*; Hu et al., 2012). In shellfish, it has been isolated from oysters (*Crassostrea rhizophorea*; Evangelista-Barreto et al., 2006), snails (Roger et al., 2012; Talagrand-Reboul et al., 2017), Yesso scallop (*Patinopecten yessoensis*; De Silva et al., 2019), shrimps (*Litopenaeus vannamei*; De Silva et al., 2018), cockles (*Tegillarca granosa*; Dahanayake et al., 2020), and clam (*Ruditapes philippinarum*; Dahanayake et al., 2019). In Norwegian markets, it has been isolated from retail foods such as sushi, oysters, and scallops (Hoel et al., 2017; Lee et al., 2021), while in Korean markets it has also been isolated from frozen shrimps, clams, and Yesso scallop (De Silva et al., 2018, 2019; Dahanayake et al., 2019, 2020). It has also been isolated from chilled chicken in China (Wang et al., 2017; Shao et al., 2022), turkey in Germany (Shen et al., 2018), and pork and pig slaughter house in Portugal (Fontes et al., 2011). These studies show that *A*. *media* can be transmitted to humans through food, drinking water and the environment.

Although *A*. *media* has been linked to diarrhea in humans and skin ulcerations in fish (Lü et al., 2016), there is limited information describing the profile of virulence factors found in *A*. *media*. It is unknown whether *A*. *media* shares a similar composition of virulence genes with other *Aeromonas* spp. like *A*. *hydrophila*, *A*. *veronii*, and *A*. *salmonicida*. As pointed out by Guerra et al. (2007) and Bhowmick and Bhattacharjee (2018) *Aeromonas* virulence is multifactorial involving various factors like endotoxins, enterotoxins, cytotoxins, hemolysins, proteases, and adhesins. However, *A*. *hydrophila*, *A*. *veronii*, *A*. *caviae*, and *A*. *sobria* are considered as major pathogens in the genus *Aeromonas* because they account for the largest proportion of the aeromonads isolated from clinical cases unlike *A*. *media*, which is considered a minor pathogen because of fewer cases isolated from human and animal diseases. Thus, there have been more virulence factor studies done for the major *Aeromonas* pathogens than for minor pathogenic species like *A*. *media* (Piotrowska and Popowska, 2015; Rasmussen-Ivey et al., 2016; Romero et al., 2016; Gauthier et al., 2017; Talagrand-Reboul et al., 2017). However, the increasing number of cases linked to human and animal infections reported in recent years coupled with increasing isolations from retail ready-to-eat foods (Fontes et al., 2011; Wang et al., 2017; Shen et al., 2018; De Silva et al., 2019; Shao et al., 2022) indicates that *A*. *media* is emerging as an important environmental and foodborne pathogen with significant public health implications. Thus, there is need to elucidate the virulence factors of *A*. *media* isolated from different aquatic environments and host species with the view of developing effective control measures.

Antimicrobial resistance (AMR) has emerged to be an important global public health threat classified as among the top 10 global priorities by World Health Organization (2021). Multidrug resistant *Aeromonas* spp. have been isolated from different aquatic environments, animals, and retail foods (Stratev and Odeyemi, 2016; Teodoro et al., 2022). Also, *Aeromonas* spp. have been shown to carry plasmids encoding AMR and virulence genes (Tomás, 2012). Although previous studies reported the presence of AMR and virulence genes from *Aeromonas* spp. that included *A*. *media* isolated from ready-to-eat foods in Norway, the major limitation with these studies was that they used primers that targeted only a few selected genes, which did not give a global overview of all AMR genes present in bacteria genomes. Thus, in the present study, we used whole genome sequencing (WGS) to characterize all virulence and AMR genes present in *A*. *media* isolated from marine sediments collected from the Øresund Bay in Denmark. To gain a wide overview of the virulence and AMR genes found in *A*. *media* strains isolated from different geographical areas, we compared our isolate (strain SD/21–15) with genomes of 24 other isolates from Europe, North America, and South America retrieved from the National Center for Biotechnology Information (NCBI). We also compared our isolate with whole genome sequences of *A*. *hydrophila*, *A*. *veronii*, and *A*. *salmonicida* reference strains to determine the difference in the composition of virulence genes between *A*. *media* strain SD/21–15 and other *Aeromonas* spp. Our findings show that WGS is a reliable tool able to profile all AMR and virulence genes found in bacteria genomes unlike PCR based assays that only identify a few selected genes based on the primers used in the assay. Thus, we found a high similarity in the profile of AMR and virulence genes found in strain SD/21–15 with other *A*. *media* strains isolated from different host species and geographical areas in the world. Our findings show that *A*. *media* harbors several intrinsic AMR genes that could be transmissible to other bacteria species and it also harbors several virulence genes that could be responsible for its pathogenicity in different host species. We anticipate that data generated in this study will shed new insights on the role of *A*. *media* in the spread AMR genes and that it will pave way for studies aimed at elucidating the virulence mechanisms of *A*. *media* in different susceptible hosts.

## Materials and methods

### Characterization of bacteria using MALDI-TOF and sequences of The 16S rRNA gene

The *A*. *media* isolate designated as strain SD/21–15, originally isolated from marine sediments collected from Øresund in Denmark in 1992 (Andersen and Sandaa, 1994), was retrieved from the −80°C freezer and cultured in tryptose soy broth (TSB) followed by incubation at 10°C for 5–7 days. The isolate was previous classified as *Aeromonas* spp. (Andersen and Sandaa, 1994). The bacteria initially grown in TSB was later cultured on blood agar plates by incubation at 10°C for 5–7 days for individual colony purity followed by characterization using the Matrix-Assisted Laser Desorption/Ionization-Time Of Flight (MALDI-TOF) mass spectrometry (MS; Singhal et al., 2015). The purified bacteria confirmed by MALDI-TOF were used for DNA extraction using the DNA extraction kit based on the manufacturer’s protocol (Qiagen, Germany). Species identification and confirmation was carried out by PCR amplification of the 16S rRNA gene using the universal primers 27F and 1492R (Kuncham et al., 2017).

### Testing of antimicrobial resistance using disk diffusion assay

The antibiotic disk experiment was carried out based on the Clinical and Laboratory Standards Institute (CLSI; Cockerill et al., 2012) guidelines to determine the susceptibility or resistance of bacteria to antibiotic treatment (Kahlmeter et al., 2006). The *A*. *media* isolate from Øresund in Denmark (Andersen and Sandaa, 1994) was tested for antibiotic resistance using the Kirby-Bauer disk diffusion assay (Joseph et al., 2011) using commercially available antibiotic discs (Neo-SensitabsTM, Rosco). Antibiotics used in the disk diffusion test were Ciprofloxacin (CIPR—5 μg), Erythromycin (Ery—15 μg), Gentamycin (GEN—10 μg), Ampicillin (AMP—10 μg), Cefoxitin (CFO—30 μg), Cephalothin (CEP—30 μg), Nitrofurantoin (NI—300 μg), Penicillin (PEN—10 μg), Tetracycline (TET—30 μg), Trimethoprim (TRIM—5 μg), Colistin (CO—150 μg), Sulfonamide (SULFA—240 μg), Amoxicillin (AMOXY—30 μg), Rifampicin (RIF—5 μg). The bacteria cultured overnight was diluted to 0.5 McFarland at a concentration of 10^8^ CFU/ml and was spread on the surface of the Muller Hinton agar using sterile cotton swabs (Saffari et al., 2016). The antibiotics discs were put on the plate containing the bacterial lawn. This was followed by incubation at 10°C for 5–7 days. Afterward, antibiotic susceptibility and resistance was measured based on the manufacturer’s instruction (Neo-SensitabsTM, Rosco).

### Bacterial genomic DNA extraction and quality control analysis

Genomic DNA (gDNA) was extracted from *A*. *media* strain SD/21–15 isolate using the MagAttract® HMW DNA kit based on manufacturer’s protocols (Qiagen, Germany) (Becker et al., 2016). A concentration of 2 × 10^9^ CFU/ml freshly grown *A*. *media* strain SD/21–15 was centrifuged in 2 ml Eppendorf tubes, and pellets were resuspended in 180 μl ATL buffer followed by adding 20 μl Proteinase K to each tube. This was followed by incubation at 56°C in an Eppendorf thermomixer for 30 min. Afterward, 4 μl RNase was added to each tube followed by pulse vortexing and adding 15 μl of MagAttract Suspension G and 280 μl Buffer MB to each vial (Tarumoto et al., 2017). Next, the suspension from each tube was transferred onto a MagAttract holder followed by mixing for 60 s on an Eppendorf thermomixer. Magnetic beads containing gDNA were separated on the MagAttract magnetic rack for 60 s. Supernatants were removed without disturbing the beads and were washed twice using MW1 and PE buffer (Becker et al., 2016; Tarumoto et al., 2017). Thereafter, the remaining suspension from each vial was removed by rinsing the beads with 1 ml distilled water twice. The gDNA was harvested by eluting in 100 μl buffer EB while the purity of the gDNA was assessed using the NanoDrop (Thermo fisher, United States) followed by gel electrophoresis using 1% agarose. The harvested gDNA was quantified using the Qubit double-stranded DNA (dsDNA) high-CHS kit based on the manufacturer’s instructions (Life Technologies Inc., Carlsbad, CA, United States; Guan et al., 2020).

### Library preparation and sequencing

The sequence library for *A*. *media* strain SD/21–15 was prepared using the paired end DNA libraries using the Nextera DNA Flex Tagmentation (Illumina Inc. San Diego, CA, United States; Gaio et al., 2021) while the Illumina library was quantified using the Qubit® DNA HS Assay Kit in a Qubit fluorometer (Thermo Fisher Scientific, Waltham, MA, United States). Agilent HS DNA Kit (Agilent Technologies, CA, United States) based on the Agilent 2,100 Bioanalyzer System was used to check the size of library fragments. Illumina MiSeq (Illumina Inc., United States) was used for sequencing using V3 reagent kits using paired-end read length of 2 × 300 bp as previously described (Kaspersen et al., 2020). Bioinformatic analysis was done using the online Galaxy platform^1^ version 21.05. Quality of both forward and reverse raw reads was analyzed using the FastQC Version 0.11.9 software (Bioinformatics, 2011). Adapters and low-quality reads from paired end sequences were removed using Trimmometric version 0.38.1 (Bolger et al., 2014). Afterward, the resulting paired-end sequence reads were *de novo* assembled into contigs using A5-miseq assembler (Coil et al., 2015). Quality read sequence contigs with 33–91 k-mers were assembled using SPAdes v. 3.12.0 (Bankevich et al., 2012). Genome annotation was made using the prokaryotic genome annotation pipeline (PGAP; Tatusova et al., 2016) from NCBI while annotation was done using Prokka (Seemann, 2014).

### Prediction of average nucleotide identity and virulence genes

In addition to the *A*. *media* strain SD/21–15 whole genome sequence (WGS), we retrieved 24 WGSs of *A*. *media* isolates from the NCBI database obtained from different host species and environmental samples from Asia, Europe, and North America (Table 1). It is noteworthy that although *A*. *media* strain SD/21–15 was isolated in 1992 when it was classified as *Aeromonas* spp. (Andersen and Sandaa, 1994), the 24 genomes retrieved from the NCBI database covered the period 2013–2022 because there were no whole genome sequences of *A*. *media* prior to 2013 found in the NCBI database. The Galaxy platform using abricate v. 1.0.1 was used to identify genes of the virulence factors of pathogenic bacteria (VFDB; Seemann, 2014, 2020; Chen et al., 2005) of which the threshold for virulence-gene identification using the VFDB was set at 80%. On the other hand, the Average Nucleotide Identity (ANI) of all 25 *A*. *media* genomes was analyzed using the online Galaxy Europe^2^ using FastANI v. 1.3. *Aeromonas media* strain MC64 from the Chinese hospital (CP047962.1) was used as a reference to calculate the ANI of all the 25 *A*. *media* genomes (Table 1). The threshold for FastANI was set at 90% based on pairwise sequence mapping (Jain et al., 2018) while heatmap based on calculated ANI for all 25 *A*. *media* genomes were generated using the package heatmap in the R studio v 4.0.4 statistical software with online Orion NMBU software.^3^

**Table 1:** Genome data of *Aeromonas media* strains used in the study.

No	Strain name	Country	Sources	Year	Level	Size (Mb)	GC%	Scafold	Genes	Proteins	Accession No.
1	TR3_1	China	Waste water	2021	Complete genome	4,531	61	1	4,193	3,954	CP075564.1
2	SD/21–15	Denmark	Marine sediment	2022	Contig	4,889	59	214	4,663	4,444	JAJVCY000000000
3	ARB13	Japan	River water	2014	Contig	4,612	61	180	4,330	4,124	JRBF00000000.1
4	ARB20	Japan	River water	2013	Contig	4,6	60.5	185	4,337	4,126	JRBG00000000.1
5	CECT 4232	USA		2013	Contig	4,5	61	329	4,299	4,043	CDBZ00000000.1
6	NXB	China	Chicken meat	2017	Scaffold	4,5	61	131	4,199	4,001	NXBV00000000.1
7	BAQ071013-132	USA	Perch	2019	Scaffold	4,7	61	165	4,394	4,174	NKWW00000000.1
8	BAQ071013-115	USA	Perch	2019	Scaffold	4,6	62	89	4,252	4,080	NKWY00000000.1
9	MC64	China	Hospital	2017	Complete genome	5	60	1	4,680	4,239	CP047962.1
10	T0.1–19	China	Sludge	2016	Complete genome	4,9	60	1	4,612	4,248	CP038441.1
11	R1-18	China	Sludge	2016	Complete genome	4,7	61	1	4,450	4,006	CP038443.1
12	T5-8	China	Sludge	2016	Complete genome	4,8	60.5	1	4,502	4,131	CP038444.1
13	R25-3	China	Sludge	2016	Complete genome	4,9	60.6	1	4,547	4,233	CP038445.1
14	R50-22	China	Sludge	2016	Complete genome	5,1	60	1	4,767	4,430	CP038448.1
15	R1-26	China	Biofilm reactor	2018	Complete genome	4,7	60.5	1	4,361	4,069	CP043579.1
16	WP7-W18-ESBL-02	Japan	Waste water	2020	Complete genome	4,8	61	1	4,424	4,165	AP022188.1
17	E31	China	water	2021	Complete genome	5,3	60	1	4,953	4,526	CP067417.1
18	CN17A0010	China	Human stool	2021	Contig	4,6	62	18	4,220	4,025	JAEHIH000000000.1
19	Colony414	Thailand	food	2021	Complete genome	4,7	62.5	1	3,934	3,535	CP070623.1
20	D180	Spain	Fish	2021	Contig	4,5	61.5	52	4,179	3,936	JAGDES000000000.1
21	ATCC 33907	Spain	River water	2021	Contig	4,5	61	199	4,227	4,001	JAGDEO000000000.1
22	Z1-6	China	Human	2018	Scaffold	4,5	61	131	4,209	4,008	UETL00000000.1
23	KLG6	UK	River	2019	Contig	4,5	61	454	4,430	3,961	CAAKNK000000000.1
24	INSAq193	Portugal	Fish	2022	Scaffold	5,2	60	532	5,101	4,704	JAKCNH000000000.1
25	WS	China	Water sample	2014	Complete genome	4,8	60.5	1	4,452	4,089	CP007567.1

### Prediction of antimicrobial resistance genes and mobile genetic elements

A total of 25 *A*. *media* whole genome sequences were used for identification of AMR genes, plasmids and transposons. Staramr version 0.7.2 (Tran et al., 2021) and ABRicate version 1.0.1 (Seemann, 2014, 2020) were used for identification of antibiotic resistance genes in the Comprehensive Antimicrobial Resistance Database (CARD) software (Alcock et al., 2020) of which the CARD identification threshold for AMR-genes was set at 80%. Identification of plasmids in bacterial genomes was done using Plasmidfinder v 2.0 (Ullah et al., 2020) with the threshold for plasmid identification set at 80%. Proksee software^4^ was used to generate circular maps of all 25 *A*. *media* genomes and plasmids online.

## Results

### Whole genome sequencing and phylogenetic analysis

**Figure 1:**
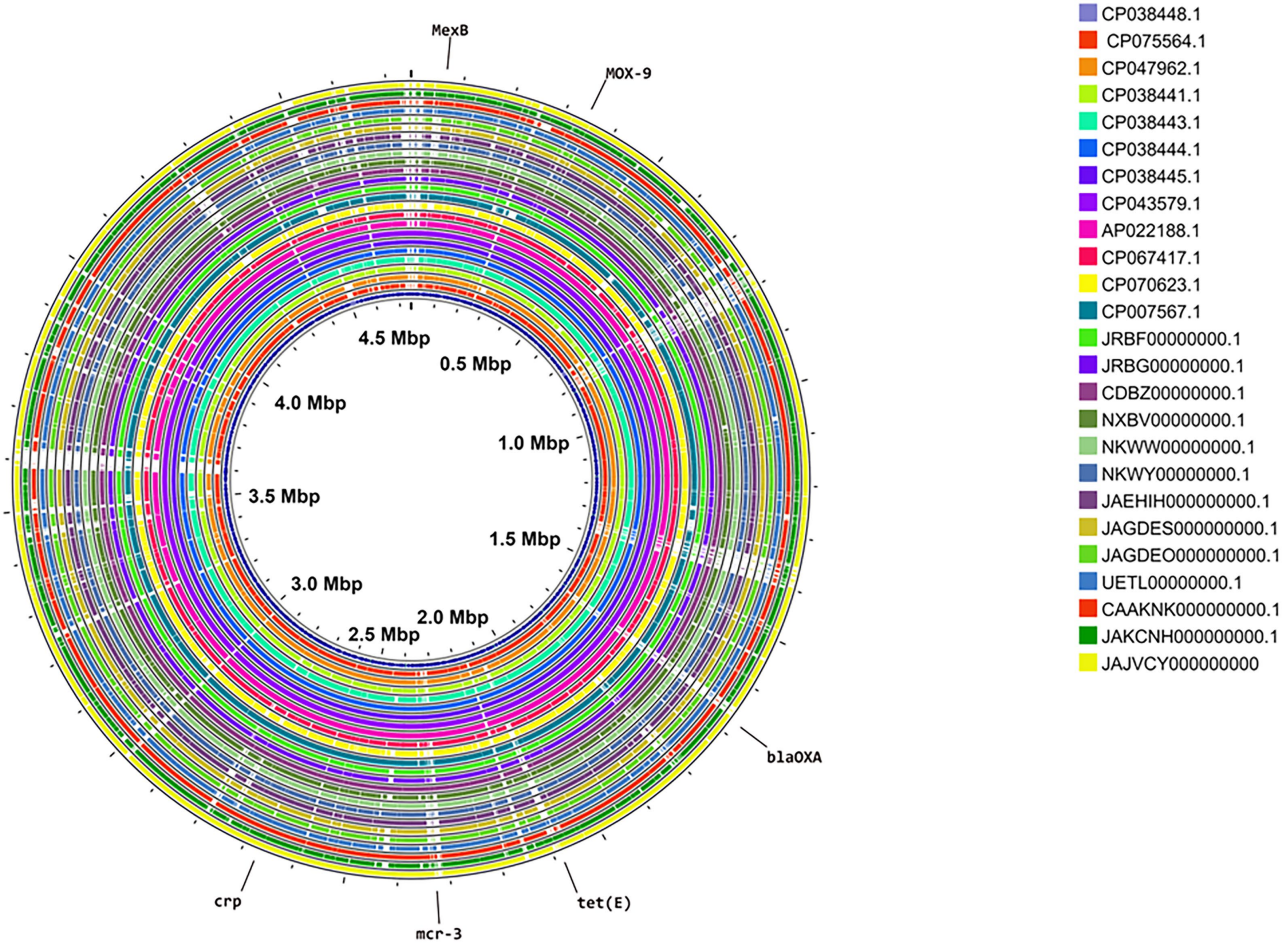
Circular map showing a comparison of the genome of *Aeromonas media* strain SD/21–15 together with genomes retrieved from the National Center for Biotechnology Information (NCBI) public databank obtained from different host species and environmental samples from different geographical areas in the world (See Table 1). Note strain SD/21–15 (JAJVCY000000000; outermost) shows a complete circular map similar with other 25 *A*. *media* strains. Figure 1 was created in Proksee (https://proksee.ca/).

The *A*. *media* genomes retrieved from the NCBI databank were from Asia, Europe, and North America with the majority coming from Asia (Table 1). Thus, we did not find whole genome sequences of *A*. *media* isolates from Africa, Central, and South America in the public database. The genome size varied between 4.5 and 5.2 Mb while the GC content varied between 59 and 62.5% for all isolates (Table 1). The number of genes detected varied between 3,934 and 5,101 while the number of proteins varied between 3,535 and 4,704. Apart from the archived strain SD/21–15 obtained from marine sediments in Øresund in Denmark in 1992, all *A*. *media* strains used were isolated from the period 2013 to 2022 indicating that they were isolated in the last decade. Other details that include strain names, country of origin, and accession numbers are shown in Table 1. The circular map showing all *A*. *media* genomes used shows that strain SD/21–15 had a complete genome comparable with other *A*. *media* isolates obtained from different host species and the environment (Figure 1). Equally, phylogenetic analysis showed high similarity (>94%) of strain SD/21–15 with other *A*. *media* isolates from different host species and environments.

### Average nucleotide identity and heatmap

**Figure 2:**
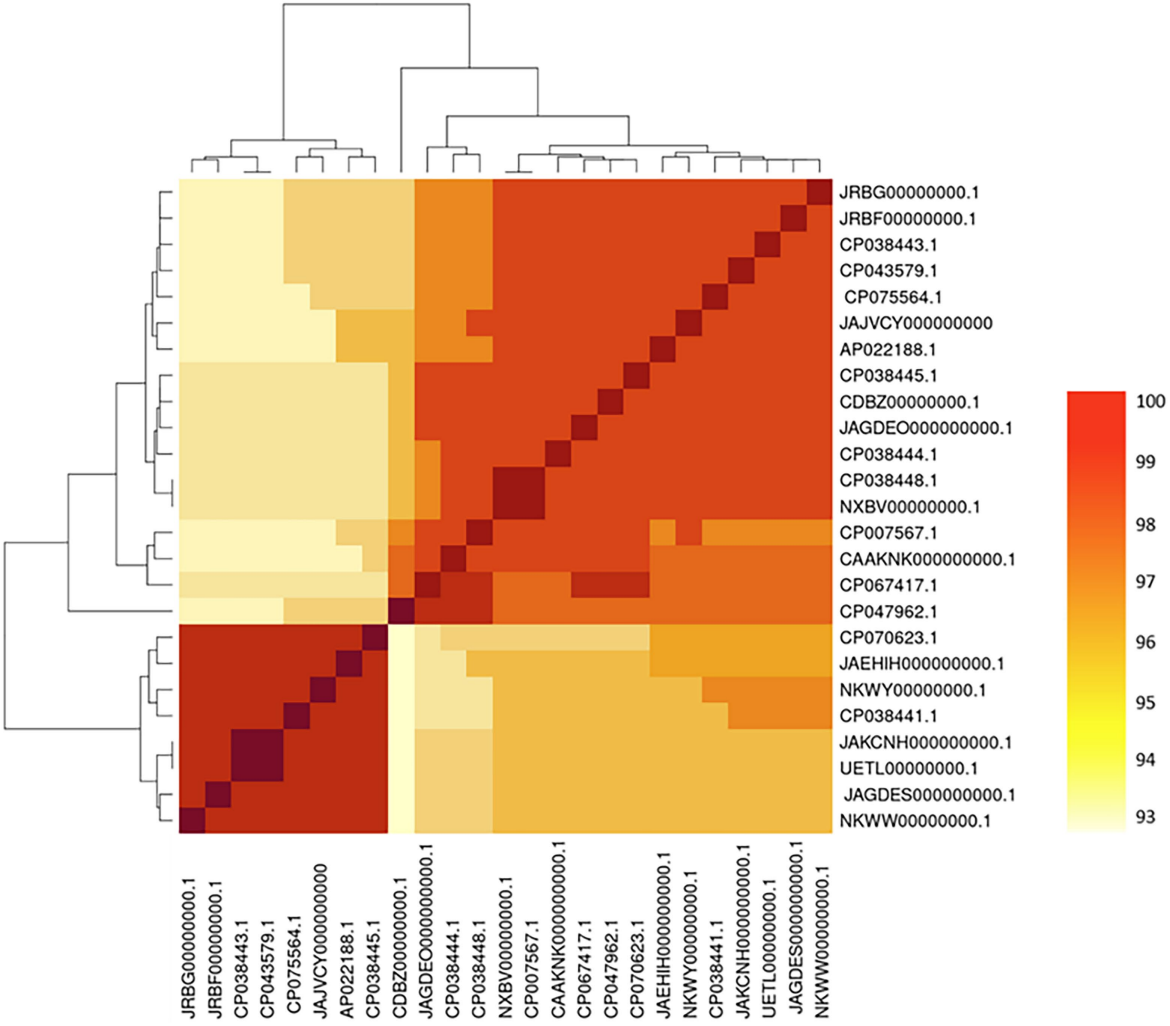
Heatmap/cladogram combined with phylogenetic comparison of *Aeromonas media* strain SD/21–15 isolated from an aquatic environment in Denmark with other strains retrieved from the National Center for Biotechnology Information (NCBI) public databank isolated from different host species and environments in the world. Note the high similarity among all *25 A*. *media* strains with homology varying between 93 and 100%.

The ANI phylogenetic analysis showed high similarity (>94%) of all *A*. *media* isolates despite emanating from different host species and geographical areas (Figure 2). The ANI of *A*. *media* strain SD/21–15 was >97% similar with the Chinese hospital strain MC64 (CP047962.1) used as a reference. The ANI phylogenetic tree clustered all 25 isolates into two groups, of which group-I comprised of 17 isolates with >97% similarities that included isolates from Denmark (SD/21–15), Japan (ARB13, ARB20, and WP7-W18-ESBL-02), China (T0.1–19, R1-26, and E31), United States (CECT 4232), Spain (ATCC 33907), and United Kingdom (KLG6; Figure 2). On the other hand, group-II comprised seven isolates with >93% similarities consisting of isolates from United States (BAQ071013-132 and BAQ071013-115), Spain (D180), Portugal (INSAq193), Thailand (Colony414), and China (CN17A0010 and Z1-6).

### Virulence factors

**Figure 3:**
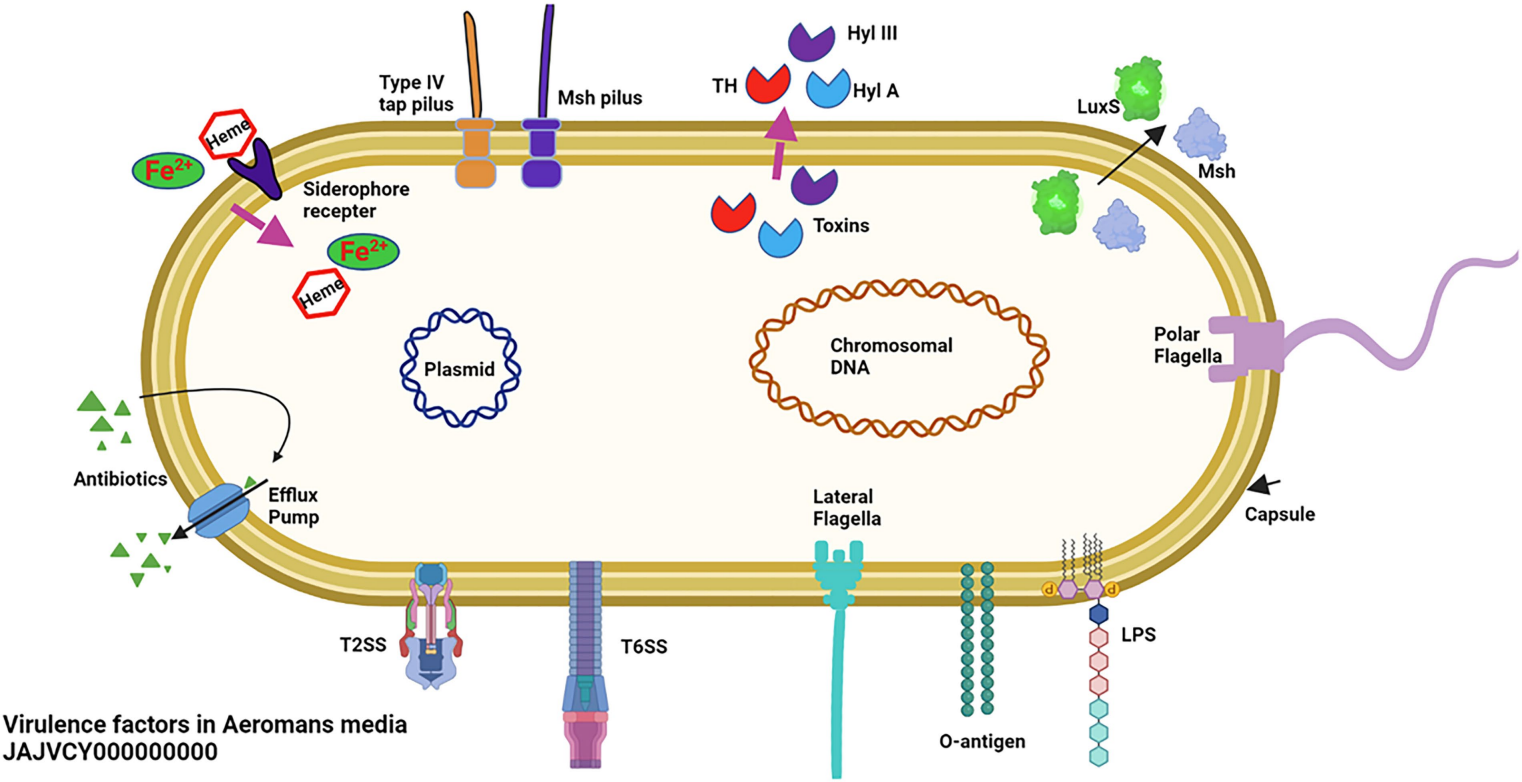
Schematic diagram of virulence genes on *Aeromonas media* included investigated showing (i) adherence proteins consisting of the *Msh* and type tap IV pili as well as the polar and lateral flagella, (ii) iron acquisition components comprising of the ferric uptake regulator (*Fur*), heme, and siderophore proteins, (iii) secretion system consisting of T2SS and T6SS, (iv) hemolysins consisting of hemolysin A (*hylA*), hemolysin III (*hylIII*), and thermostable heat (TH) protein, (v) biofilm and quorum sensing components consisting of *S*-ribosylhomocysteinase (*LuxS*) and MQS, (iv) Immune proteins consisting of capsule, lipopolysaccharide (LPS), and somatic O-antigen. In addition, schematic diagram shows *A*. *media* components associated with antimicrobial resistance (AMR) components consisting of (vii) efflux pumps and (viii) plasmid. Figure 3 was created in BioRender.com (https://biorender.com/).

The virulence factors examined comprised of six elements, namely; (i) adherence and motility, (ii) immune evasion, (iii) secretions system, (iv) toxins, (v) iron acquisition, and (vi) biofilm formation together with quorum sensing (Figure 3; Table 2).

**Table 2:** Comparison of virulence genes in *Aeromonas media* sequences.

**Strain name**	**Adherence and motility**				**Immune evasion**	**Secretion system**						**Toxins**					**Iron acquisition**			**Biofilm and quorum sensing**	
	**Msh pilus**	**Polar flagella**	**Tap type IV pili**	**Lateral flagella**	**Capsule**	**T2SS**	**T3SS**	**T6SS**				***hylHlA***	***hylIII***	***TSH***	**AerA**	**RTX toxin**	**Ferric uptake**	**Siderophore synthesis**	**Heme uptake**	**LuxS**	**mshA-Q**
								**vgrG1**	**vgrG3**	**hcp**	**ats**										
TR3_1																					
**SD/21–15**																					
ARB13																					
ARB20																					
CECT 4232																					
NXB																					
BAQ071013-132																					
BAQ071013-115																					
MC64																					
T0.1–19																					
R1-18																					
T5-8																					
R25-3																					
R50-22																					
R1-26																					
WP7-W18-ESBL-02																					
E31																					
CN17A0010																					
Colony414																					
D180																					
ATCC 33907																					
Z1-6																					
KLG6																					
INSAq193																					
WS																					

Blue, presence of gene, white/blank, absence of the gene.

#### Adherence, motility proteins, and immune evasion genes

The adherence and motility genes detected were classified into four groups namely the (i) Msh pilus, (ii) Tap type IV pili, (iii) polar flagellar, and (iv) lateral flagella (Table 2). The Msh pilus, polar flagellar, and Tap type IV pili genes were detected in all 25 *A*. *media* strains including strain SD/21–15 while the lateral flagella genes were only found in strains BAQ071013-132 and BAQ071013-1115 isolated from perch in the United States as well as strain D180 isolated from fish in Spain (Table 2). Comparison of strain SD/21–15 with other *Aeromonas* spp. showed that it had all genes that form the Flp type IV and polar flagella proteins similar with the *A*. *hydrophila* (ATCC 7966), *A*. *veronii* (B565), and *A*. *salmonicida* (A449) reference strains (Supplementary Table S1). However, it only had 15 genes that form the Tap type IV pili unlike *A*. *veronii* (B565), *A*. *hydrophila* (ATCC 7966), and *A*. *salmonicida* (A449) reference strains that had 20, 22, and 23 proteins, respectively. Our findings also show that *A*. *media* SD/21–15 strain did not have type I fimbriae genes found in *A*. *veronii* (B565), *A*. *hydrophila* (ATCC 7966), and *A*. *salmonicida* (A449) reference strains. In addition, genes that form the lateral flagella were only detected in *A*. *salmonicida* (A449) but not in *A*. *media* strain SD21/01–15 and the other *Aeromonas* reference strains (Supplementary Table S1). On the contrary, the polar flagella genes were detected in all four *Aeromonas* spp. examined although *A*. *hydrophila* (ATCC 7966) and *A*. *media* strain SD/21–15 had more genes that form the polar flagella than *A*. *veronii* (B565) and *A*. *salmonicida* (A449) reference strains (Supplementary Table S1).

#### Capsule and immune evasion genes

Our findings show that only eight of the 25 isolates examined had capsules and those included the strains SD/21–15, ARB13, CN17A0010, D180, ATCC 33907, Z1-6, KLG6, and INSAq193 (Table 2). A comparison of strain SD/21–15 with other *Aeromonas* spp. showed that only strain SD/21–15 had a capsule, and no capsule genes were detected in the genomes of *A*. *hydrophila* (ATCC 7966), *A*. *veronii* (B565), and *A*. *salmonicida* (A449; Supplementary Table S1). Other immune evasion genes detected in *A*. *media* strain SD/21–15 include the nitrate reductase (*narH*), antiphagocytosis capsule (*wzb*), serum resistance LPS (*rfb*), and stress adaptation catalase peroxidase (*karG*) genes (Supplementary Table S1).

#### Secretion system

Although we investigated the presence of all secretory systems, our findings show that only the type II secretory system (T2SS) was detected in all 25 *A*. *media* (Table 2). Comparative analysis showed that strain SD/21–15 had 14 of the 15 T2SS genes ranging from *exeA* to *exeM* with the exception of *exeN* while *A*. *hydrophila* (ATCC 7966), *A*. *veronii* (B565), and *A*. *salmonicida* (A449) had all 15 genes from *exeA* to *exeN* (Supplementary Table S1). On the contrary, the type III secretory system (T3SS) was not detected in all 25 *A*. *media* genomes, and it was not detected in *A*. *hydrophila* (ATCC 7966), *A*. *veronii* (B565), and *A*. *salmonicida* (A449) reference strains. As for the type VI secretory system (T6SS), only three isolates had the genes *vrG1*, *vgrG3*, *hcp*, and *ats* genes in their genomes while most strains only had two of these genes detected (Table 2). Comparison of strain SD/21–15 with other *Aeromonas* spp. showed that only *A*. *hydrophila* (ATCC 7966) had all 25 genes that form the T6SS while *A*. *salmonicida* (A449) had 14 and *A*. *veronii* (B565) had none (Supplementary Table S1).

#### Hemolysin and other toxin genes

All *A*. *media* isolates had hemolysin genes namely hemolysin HlyA (*hlyA*), hemolysin III (*hlyIII*), and thermostable hemolysin (*TSH*) genes (Table 2). On the contrary, the aerolysin gene was not detected in all 25 *A*. *media* strains while the *RTX* toxin genes were only detected from Strain NXB, T0.1–19, and Z1-6 from chicken meat, sludge, and humans in China (Tables 1, 2), respectively. Comparison of strain SD/21–15 with other *Aeromonas* spp. showed that the aerolysin AerA/cytotoxic enterotoxin *aerA/act* gene was present in *A*. *hydrophila* (ATCC 7966), *A*. *veronii* (B565), and *A*. *salmonicida* (A449) reference strains but not in strain SD/21–15. Other toxin genes, such as the heat stable cytotoxic enterotoxin (*ast*) and the repeat toxins (*RTX*; *rtxA*, *rtxB*, *rtxC*, *rtxD*, *rtxE*, and *rtxH*) genes only found in *A*. *hydrophila* (ATCC 7966) were not detected in strain SD/21–15. It also lacked the extracellular hemolysin (*ahh1*) gene found in *A*. *hydrophila* (ATCC 7966) and *A*. *salmonicida* (A449). On the other hand, *hlyA*, *hlyIII*, and TH toxin genes found in strain SD/21–15 were also present in *A*. *hydrophila* (ATCC 7966), *A*. *veronii* (B565), and *A*. *salmonicida* (A449) reference strains.

#### Iron acquisition, biofilm formation, and quorum sensing genes

The biofilm and quorum sensing *luxS* and *mshA-Q* genes were present in the genomes of all *A*. *media* isolates examined (Table 2). Similarly, the iron acquisition genes consisting of the gene of ferric uptake regulator (*fur*), siderophore synthesis, and heme uptake genes were present in all *A*. *media* isolates (Table 2).

### Antimicrobial resistance

#### Phenotype characterization using the disk diffusion test

The *A*. *media* strain SD/21–15 showed multidrug resistance (MDR) to more than five antibiotics that included AMP-10 and PEN-10. They also showed resistance to CFO-30, CEP-30, TET-30, and AMOXY-30. It showed intermediate resistance for CIPR-5, ERY-15, and RIF-5 but was susceptible to Gentamycin GEN-10, NI-300, SULFA-, and TRIM-5 (Table 3) on the disk diffusion test.

**Table 3:** Antimicrobial resistance of *Aeromonas media* strain SD/21–15 based on disc diffusion test.

**Antibiotics**	**Susceptibility/Resistance**	
Ampicillin (AMP-10)	Resistant	R
Cefoxitin (CFO30)	Resistant	R
Cephalothin (CEP 30)	Resistant	R
Ciprofloxin (CIPR5)	19 (Susceptible)	S
Erythromycin (ERY15)	11 (Susceptible)	S
Gentamycin (GEN10)	24 (Susceptible)	S
Nitrofurantoin (NI300)	20(Susceptible)	S
Penicillin (PEN10)	Resistant	R
Colistin (CO150)	28 (Susceptible)	S
Sulphonomide (SULFA)	17 (Susceptible)	S
Tetracycline (TET30)	Resistant	R
Trimethoprim (TRIM5)	20 (Susceptible)	S
Amoxicillin (AMOXY)	Resistant	R
Rifampicin (RIF5)	15 (Susceptible)	S

#### Antimicrobial resistance genes

Whole genome sequence analysis showed that all 25 *A*. *media* isolates had multiple AMR genes encoded in their genomes (Table 4). Only *bla*_KPC-1_ and *bla*_TEM-1_ were detected among class A β-lactamases, of which *bla*_KPC-1_ was found in strains MC64 and E31 that were isolated from a hospital and water in China while *bla*_TEM-1_ was found in strains MC64, E31 and INSAq193 isolated from hospital, water and fish from China and Portugal, respectively. The only gene identified in the class B metallo-β-lactamases (MBL) was *cphA7* found in strains R1-18 and R1-26 isolated from sludge and biofilm reactors in China, respectively. The class C β-lactamase group was dominated by *bla*_MOX-9_ found in all 25 *A*. *media* isolates while *bla*_CMY-8b_ was only detected in strain SD/21–15. Equally, class D was dominated by *bla*_OXA-427_ found in all 25 *A*. *media* strains while *bla*_OXA-1_ was only found in strain R50-22 and *bla*_OXA-10_ in strain WP7-W18-ESBL-02. Outside the β-lactamase, the dominant AMR genes detected were *CRP* and *MCR* that were present in all 25 *A*. *media* isolates followed by *MCR-3* and *MCR-3*.*6* that were detected in eight, and *sul1* from five isolates. Other AMR genes detected from different *A*. *media* strains are shown in Table 4.

**Table 4:** Antimicrobial resistance and efflux pump proteins detected in the *Aeromonas media* strains.

		**Beta lactamase**						**Other AMR genes**										**Efflux pump proteins**	
**No**	**Strain name**	**Class A**		**Class B MBL**	**Class C**		**Class D**												
1	TR3_1				MOX-9 (98.35)		OXA-427 (98.36)		CRP (78.41)	MCR-7.1 (72.31)					QnrS2 (100)			tet E (99.92)	MexB (73.00)
2	SD/21–15				MOX-9 (98.35)	CMY-8b	OXA-427 (98.74)		CRP (78.41)	MCR-7.2 (73.57)								tet E (99.95)	MexB (72.73)
3	ARB13				Mox-9 (98.09)		OXA-427 98.74		CRP (78.41)	MCR-7.1 (72.53)						ugd (70.40)			MexB (72.67)
4	ARB20				MOX-9 (89.09)		OXA-427 (98.74)		CRP (78.41)	MCR-7.1 (72.53)						ugd (70.40)			MexB (72.67)
5	CECT 4232				MOX-9 (99.91)		OXA-427 (97.99)		CRP (78.41)	MCR-7.1 (73.19)	MCR-3 (83.77)	MCR-3.6 (99.88)		vatF (71.04)					MexB (72.47)
6	NXB				MOX-9 (85.62)		OXA-427 (89.31)		CRP (78.73)	MCR-7.1 (73.29)	MCR-3 (84.26)	MCR-3.6 (96.55)						tet E (95.28)	MexB (72.73)
7	BAQ071013-132				mox-9 (85.96)		OXA-427 (89.43)		crp (78.56)	MCR-7.1 (73.22)									MexB (72.40)
8	BAQ071013-115				MOX-9 (88.29)		OXA-427 (89.31)		CRP (77.94)	MCR-7.1 (73.54)				vatF (71.92)					MexB (72.82)
9	MC64 (Plasmid)	KPC-1 (100)	TEM-1		MOX-9 (98.44)		OXA-427 (99.12)		CRP (78.41)	MCR-7.1 (73.38)				AAC(3)-Iid (99.88)	mphA (100)	ugd (71.24)			MexB (72.70)
10	T0.1–19				MOX-9 (85.60)		OXA-427 (88.68)		CRP (78.73)	MCR-7.1 (73.43)									MexB (72.41)
11	R1-18			cphA7 (94.12)	MOX-9 (99.91)		OXA-427 (98.99)		CRP (78.41)	MCR −7.1 (72.69)			sul1 (100)	ANT (3)-II a(99.07)	aadA16 (99.29)	catB (100)	dfrB4 (100)	tet E (95.19)	MexB (72.48)
12	T5-8				Mox-9 (99.90)		OXA-427 (97.99)		CRP (78.73)	MCR-7.1 (73.42)	MCR-3 (83.77)	MCR-3.6 (99.88)		vatF (71.04)				tet E (99.92)	MexB (72.73)
13	R25-3				Mox-9 (99.90)		OXA-427 (97.99)		CRP (78.41)	MCR-7.1 (73.19)	MCR-3 (83.77)	MCR-3.6 (99.88)		vatF (71.04)				tet E (99.92)	MexB (72.73)
14	R50-22 (Plasmid)				MOX-9 (99.90)		OXA-427 (97.99)	OXA-1 (100)	CRP (78.41)	MCR-7.nnnnn(73.19)	MCR-3 (99.89)	MRC 3.6 (99.88)	sul1 (100)	AAC (6)-Ib-cr (100)	arr-3 (100)	catB (100)	mphE (100) msrE (100)	tet E(99.92)	MexD (80.230)
15	R1-26			cphA7 (94.12)	MOX-9 (99.91)		OXA-427 (98.99)		CRP (78.41)	MCR-7.1 (72.97)			sul1 (100)	ANT(3)-Iia	tet A (100)	catB3 (100)	dfrB4 (100)		MexB (72.35)
16	WP7-W18-ESBL-02				MOX-9 (98.09)		OXA-427 (98.99)	OXA-10	CRP (78.57)	MCR-7.1 (72.96)			sul1 (100)	ANT (3)-IIa	tet A (100)	catB3 (100)	dfrB4 (100)		MexB (72.92)
17	E31	KPC-1 (99.89)	TEM-1 (99.89)		MOX-9 (98.44)		OXA-427 (98.99)		CRP (78.41)	MCR-7.1 (73.38)				AAC(3)-Iid (99.88)		mphA (100)		tet E(96.95)	MexB (72.62)
18	CN17A0010				MOX-9 (88.29)		OXA-427 (89.56)		CRP (97.95)	MCR 7.1 (73.51)				vatF (71.07)					MexB (72.75)
19	Colony414				MOX-9 (89.21)		OXA-427 (89.38)		CRP (77.94)	MCR-7.1 (73.02)									MexB (72.36)
20	D180				MOX-9 (86.22)		OXA-427 (89.06)		CRP (78.41)	MCR-7.1 (73.48)									MexB (72.31)
21	ATCC 33907				MOX-9 (99.74)		OXA-427 (97.99)		CRP (78.41)	MCR-7.1 (73.19)	MCR3 (83.77) MCr	MCR-3.6 (99.88)							MexB (72.47)
22	Z1-6				MOX-9 (99.74)		OXA-427 (89.31)		CRP (78.73)	MCR-7.1 (73.29)	MCR-3 (84.26)	MCR3.6 (96.55)						tet (E) (95.28)	MexB (72.73)
23	KLG6				MOX-9 (98.44)		OXA-427 (98.99)		CRP (78.41)	MCR-7.1 (73.56)					tet A (100)				MexB (72.92)
24	INSAq193		TEM-1		MOX-9 (85.87)		OXA-427 (89.12)		CRP (78.57)	MCR 7.1 (71)		acrD (98.81)	sul2 (99.63)	APH (3)-Ib	QnrB5 (99.12)		dfrA5 (98.73)	tet E (99.92)	
25	WS				MOX-9 (98.44)		OXA-427 (98.99)		CRP (78.09)	MCR-7.1 (73.45)								tet E (95.19)	MexB (72.70)

#### Multidrug resistance proteins

Our findings show that different genes encoding multidrug resistance proteins were detected from each of the 25 *A*. *media* isolates. Among these *mexB* was detected in 24 of the total 25 *A*. *media* isolates while *tetE* was detected in 11 and *Mcr3* in seven of the 25 isolates (Table 4). Other multidrug efflux pump proteins detected included *vatF*, *catB*, *mphA*, *mphE*, *msrE*, *arr-3*, and *ugd tet*(*E*) (Table 4).

#### Mobile genetic elements

**Figure 4:**
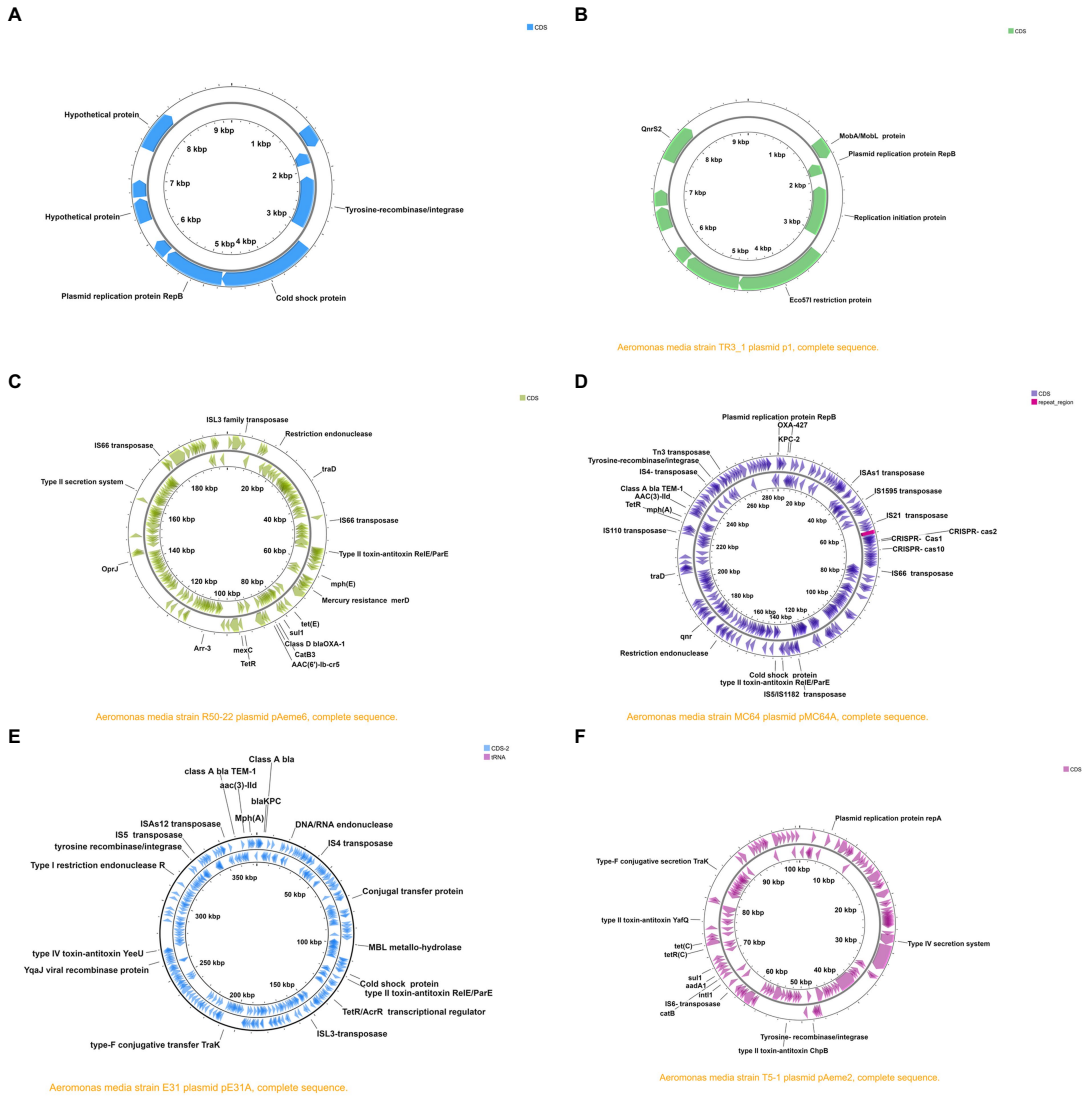
Circular maps of plasmids (i) pSD/21–15 isolated from Denmark **(A)**, (ii) pTR3_1 waste water treatment from United States **(B)**, (iii) pR50_22 from a sludge in China **(C)**, (iv) pMC64 from a hospital in China **(D)**, (v) pE31 from water in China **(E)**, and (vi) pT5-1 from a sludge in China **(F)**. Note that the location of virulence factors, antimicrobial resistance (AMR) genes, transposases, efflux pumps, secretion system, and other genes are shown in the circular map for each the plasmid.

Components of the mobile genetic elements (MBE) identified consisted of the transposases, integrases, recombinases, and plasmids (Tables 5, 6; Figure 5). Our findings show that strain D/21–15 had more transposons detected than the other strains (Table 5). Although the Tn3 family of transposons was detected in several strains, the insertion sequence (IS) class of transposons was the most dominant in all *A*. *media* isolates. Although integrase was detected in all 25 isolates only six isolates had all four components of the recombinases comprising of the recombinase family protein, tyrosine recombinase XerC, recombinase RecA, and site-specific tyrosine recombinase XerD while most strains only had the tyrosine recombinase XerC and site specific tyrosine recombinase XerD (Table 5). Finally, only 10 isolates had plasmids of which strains MC64, R25-3, R50-22, and E31 had two plasmids each while strains TR3_1, SD/21–15, T0.1–19, T5-8, INSAq193, and WS had only one plasmid each (Table 6). We generated circular maps from six out of the 10 plasmids detected to determine whether they encoded AMR genes, transposons, and integrases. The circular map of strain SD/21–15 plasmid has no AMR genes, transposases, or integrase encoded in its genome (Figure 4A). However, the circular maps of strains TR3_1, R50-22, MC64, PE31A, and T5-1 plasmids encoded AMR genes, transposases, virulence factors, efflux pumps, recombinases, and other genes (Figures 4B–F).

**Table 5:** Transposes, integrases and recombinases detected in the *Aeromonas media* genomes.

Transposes/ Integrases gene description		JAJVCY000000001	CP047962.1	CP075564.2	JRBF00000000.1	JRBG00000000.1	CDBZ00000000.1	NXBV00000000.1	NKWW00000000.1	NKWY00000000.1	CP038441.1	CP038443.1	CP038444.1	CP038445.1	CP038448.1	CP043579.1	AP022188.1	CP067417.1	JAEHIH000000000.1	CP070623.1	JAGDES000000000.1	JAGDEO000000000.1	UETL00000000.1	CAAKNK000000000.1	JAKCNH000000000.1	CP007567.1
Transposes	DDE-type integrase/transposase/recombinase																									
	IS5/IS1182 family transposase																									
	IS1595 family transposase																									
	IS110 family transposase																									
	IS3 family transposase																									
	IS5 family transposase																									
	IS66 family transposase																									
	IS630 family transposase																									
	IS4 family transposase																									
	IS21 family transposase																									
	IS30 family transposase																									
	IS200/IS605 family transposase																									
	IS256 family transposase																									
	Tn3 family transposase																									
	IS1634 family transposase																									
Integrase	Site-specific integrase																									
	Integrase																									
	Tyrosine-type recombinase/integrase																									
Recombinase	Recombinase family protein																									
	Tyrosine recombinase XerC																									
	Recombinase RecA																									
	Site-specific tyrosine recombinase XerD																									

Blue=presence of the gene, while/blank=absence of the gene.

**Table 6:** Plasmids detected in the *Aeromonas media* whole genome sequences.

**No**	**Strain name**	**Accession number**	**Plasmid-1**	**Plasmid-2**
1	TR3_1	CP075564.1	CP075565.1 (qnrS) (9,182 bp)	
2	SD/21–15	JAJVCY000000000	Contig 81, 9,295 bp	
3	ARB13	JRBF00000000.1		
4	ARB20	JRBG00000000.1		
5	CECT 4232	CDBZ00000000.1		
6	NXB	NXBV00000000.1		
7	BAQ071013-132	NKWW00000000.1		
8	BAQ071013-115	NKWY00000000.1		
9	MC64 (Plasmid)	CP047962.1	CP047963.1 (283,486 bp)	CP047964.1 (24,044 bp)
10	T0.1–19	CP038441.1	CP038442 (2,785 bp)	
11	R1-18	CP038443.1		
12	T5-8	CP038444.1	CP061478.1 (100,709 bp)	
13	R25-3	CP038445.1	CP038446.1 (190,780 bp)	CP038447.1 (4,795 bp)
14	R50-22 (Plasmid)	CP038448.1	CP038449.1 (198,927 bp)	CP038450.1 (199,818 bp)
15	R1-26	CP043579.1		
16	WP7-W18-ESBL-02	AP022188.1		
17	E31	CP067417.1	CP067418.1 (373,184 bp) KPC,	CP067419.1 (15,349 bp)
18	CN17A0010	JAEHIH000000000.1		
19	Colony414	CP070623.1		
20	D180	JAGDES000000000.1		
21	ATCC 33907	JAGDEO000000000.1		
22	Z1-6	UETL00000000.1		
23	KLG6	CAAKNK000000000.1		
24	INSAq193	JAKCNH000000000.1	JAKCNH010000351.1	
25	WS	CP007567.1	CP007568.1 (11,276 bp)	

## Discussion

In this study, we have shown that strain SD/21–15 has several virulence and AMR genes similar to those found in other *Aeromonas* spp. Although we did not find whole genome sequences of *A*. *media* from Africa, Central, and South America in the public databanks, the 25 strains used in this study show a wide geographical distribution covering North America, Europe, and Asia. The absence of whole genome sequences of *A*. *media* from Africa and South America in the NCBI database is unknown whether this is due to lack of studies or resources for WGS of *A*. *media* in these continents. In terms of host distribution, the 25 isolates used covered a wide range of hosts from humans, fish, and chickens while environmental samples were from rivers, sludge, water treatment facility, hospital, biofilm reactors, and marine environments. As for the time span covered, the isolates used covered the period 2013–2022, with the exception of strain SD/21–15 isolated in 1992, because we did not find whole genome sequences of *A*. *media* deposited in the NCBI database prior to 2013. Note that strain SD/21–15 was initially classified as *Aeromonas* spp. using morphological, motility, and biochemical tests in 1992 (Andersen and Sandaa, 1994) but the WGS carried out in the present study classified the isolate as *A*. *media*. Thus, it is likely that several other isolates previously classified as *Aeromonas* spp. using morphological and biochemical tests could classified as *A*. *media* using WGS. Nonetheless, our comparison of AMR and virulence genes for *A*. *media* in the present study is based on a collection of genomes from a broad geographical distribution and wide host species using recent data. The similarity of AMR genes detected in strain SD/21–15 from marine sediments isolated in 1992 with recent isolates covering the period 2013 to 2022 is suggestive that *A*. *media* could be a hidden environmental risk carrying several intrinsic AMR genes as a source of transmission to other bacteria. The diverse host range and environmental source is suggestive that *A*. *media*, like other aeromonads, bridges the gap between the environment, aquaculture, animals and humans in the transmission of AMR genes.

The adherence of bacteria to host cells using pili and flagella is a crucial pathogenicity step in early stages of bacterial infection. The presence of genes that form *Msh* pili, tap type IV pili, and polar flagella proteins in all 25 isolates is suggestive that these proteins could be important for the adherence of *A*. *media* to host cells. This finding shows that *A*. *media* shares similar adherence proteins with other *Aeromonas* spp. where these proteins are used for intestinal adherence, colonization and biofilm formation (Canals et al., 2006; Hadi et al., 2012). However, only three isolates had the lateral flagella genes suggesting that this protein might not be obligatory for the adherence and biofilm formation in *A*. *media*. Other genes detected include *luxS* needed for biofilm formation and quorum sensing (Kozlova et al., 2008) and *mshQ* required for mannose-sensitive hemagglutinin pilus biosynthesis (Qin et al., 2014). Thus, detection of *luxS* and *mshQ* in all 25 isolates is suggestive that these proteins could be vital for biofilm and quorum sensing in *A*. *media*.

Previous studies reported presence of four (T2SS, T3SS, T4SS, and T6SS) secretory systems in *Aeromonas* spp. out of the six characterized in Gram negative bacteria (Beaz-Hidalgo and Figueras, 2013). However, only the T2SS and T6SS were detected in the *A*. *media* isolates examined in this study. Detection of all T2SS genes in all isolates may indicate that it might be required for *A*. *media* virulence. In other *Aeromonas* spp., T2SS has been linked with the presence of various proteins such as amylases, DNases, proteases, and the aerolysin-related cytotoxic enterotoxin *Act* shown to cause diarrhea (Xu et al., 1998; Sandkvist, 2001; Sha et al., 2002; Galindo et al., 2004). On the other hand, the T6SS uses the glycine repeat G (*VrgG*) and hemolysin-coregulated (*Hcp*) genes as part of the pore-forming protein to inject toxins into host cells (Bingle et al., 2008). Our findings show that only 3/25 isolates had the genes for all four proteins (VgR1, VgrG1, VgrG3, and Hcp) characterized to be crucial for T6SS virulence in *Aeromonas* spp. (Suarez et al., 2008, 2010). Interestingly, Rasmussen-Ivey et al. (2016) pointed out that T6SS is not obligatory for *Aeromonas* virulence as shown that not all hypervirulent *A*. *hydrophila* strains causing diseases in fish possess the T6SS. Similarly, it is likely that T6SS is not obligatory for the virulence of *A*. *media* given that most isolates used in this study did not have all T6SS genes. However, there is need for *in vivo* studies to validate these observations.

We detected three hemolysin genes namely *hlyA*, *hlyIII*, and *TSH* in all 25 isolates suggesting that these genes might be important for *A*. *media* virulence. Previous studies have shown that *hlyA*, *hlyIII*, and *TH* are pore forming cytotoxic enterotoxins found in different bacteria species including *Aeromonas* spp. that cause membrane damage and fluid accumulation in host cells leading to diarrhea (Agger et al., 1985; Kozaki et al., 1987; Honda et al., 1992; Baida and Kuzmin, 1996; Stanley et al., 1998; Chopra and Houston, 1999; Abrami et al., 2000; DelVecchio et al., 2002; Wang et al., 2003; Maté et al., 2014; Abdel-Fattah et al., 2017). Thus, it is likely that the diarrhea reported in humans infected by *A*. *media* might be caused by the hemolysin genes. However, we did not find the *aerA* gene in all 25 *A*. *media* isolates and yet it was present in other *Aeromonas* spp. examined (Hirono and Aoki, 1991; Wang et al., 2003). Wong et al. (1998) and Heuzenroeder et al. (1999) showed that a combination of the *hlyA*(+)*aerA*(+) double mutant significantly reduced the virulence of *A*. *hydrophila* in mice. They observed that cytotoxicity to buffalo green monkey kidney cells and hemolysis on horse blood agar were eliminated only in the double and not in the single mutants of *A*. *hydrophila*, *A*. *veronii*, and *A*. *caviae*. They also showed that only the double mutant eliminated the β-hemolysis on horse blood agar and cytotoxic activities on buffalo green monkey and Vero cells. Inactivation of the double mutant completely attenuated the virulence of *A*. *hydrophila* in mice (Heuzenroeder et al., 1999). In this study, all *A*. *media* isolates only had *hlyA* but not *aerA*. So, it is unknown whether the absence of *aerA* renders *A*. *media* isolates less pathogenic than other *Aeromonas* spp. that have the *hlyA*(+)-*aerA*(+) combination.

Iron is a vital cofactor used for various metabolic processes for the survival of bacteria in infected hosts (Ratledge and Dover, 2000; Wandersman and Delepelaire, 2004; Maltz et al., 2015). Thus, different bacteria species have devised various mechanisms for getting iron from their hosts (Byers, 1987; Calderwood and Mekalanos, 1987; Litwin and Calderwood, 1993; Morton et al., 2007, 2009). So, the uptake of iron from host cells is considered a virulence factor because of the damage impacted on the host due to iron deprivation. Common molecules used by Gram negative bacteria for iron uptake include the ferric uptake regulator (*fur*), siderophores, and heme (Barghouthi et al., 1989a,b; Litwin and Calderwood, 1993; Morton et al., 2007). Ebanks et al. (2013) showed that *fur* knockout mutants reduced the pathogenicity of *A*. *salmonicida* while Najimi et al. (2008a) showed that mutation in the hemin-binding protein caused a drastic reduction in the pathogenicity of *A*. *salmonicida* due to reduced heme uptake as a source of iron. In another study, Najimi et al. (2008b) showed that mutations in genes used for catecholate siderophore production reduced the pathogenicity of *A*. *salmonicida*. Thus, the detection of the genes encoding *fur*, siderophore, and heme in all 25 isolates is suggestive that these genes could be crucial for iron acquisition in *A*. *media* being similar to observations seen in other *Aeromonas* spp. (Byers, 1987; Najimi et al., 2008b; Ebanks et al., 2013).

A recent study by Ebmeyer et al. (2019) reported *Aeromonas* spp. as the origin of several clinically significant β-lactamases such as the CMY-1/MOX-family that include *bla*_AmpC_, *bla*_MOX-1_, *bla*_MOX-2_, and *bla*_MOX-9_. Thus, the detection of *bla*_MOX-9_ in all 25 isolates from different host species and geographical areas in the world corroborates with Ebmeyer (2021) who reported *A*. *media* as the origin of *bla*_MOX-9_. In 2017, Bogaerts et al. (2017) reported that *bla*_OXA-427_ from Enterobacteriaceae was closely related to isolates from *A*. *media*, *A*. *hydrophila*, and *A*. *sobria* as a novel emerging carbapenem-hydrolysing class D β-lactamase (CHDL) from patients in a Belgian hospital. They showed that *bla*_OXA-427_ hydrolyzed imipenem and conferred resistance to extended-spectrum cephalosporins, penicillin and carbapenems when expressed in *Escherichia coli*. Its presence in all 25 isolates emanating from North America, Europe, and Asia is suggestive that *bla*_OXA-427_ could be highly prevalent in *A*. *media* strains across the world posing the danger of being the source of *bla*_OXA-427_ transmission to humans and animals. Its higher presence among *Aeromonas* spp. than other bacterial species, support observations made by Bogaerts et al. (2017) who pointed out that CHDLs are restricted to a few bacterial genera. Detection of *CRP* and *MCR* genes in all 25 *A*. *media* shows that the presence of these genes in *A*. *media* extends across several continents while the presence of *bla*_KPC-1_ in *A*. *media* isolates is a significant finding given that infections caused by *bla*_KPC-1_ producing bacteria are extremely difficult to treat because of their multidrug resistance linked to high mortalities in humans (Sacha et al., 2009). The presence of *MexB* in all isolates is suggestive that this efflux pump could be important for transportation of genes like *bla*_MOX-9_, *bla*_OXA-427_, *crp*, and *mcr* genes found in all *A*. *media* isolates.

Although the plasmid of strain SD/21–15 had no AMR-genes in its genome, other *A*. *media* isolates had plasmids having various AMR genes that included *bla*_KPC-1_, *bla*_OXA-427_, *sul1*, *bla*_OXA-1_, and *qnr* genes. In additions, the detected plasmids had several transposases, such as Tn3, ISAs1, IS1595, and IS4 known to carry various AMR-gene cassettes (Dziewit et al., 2012; Baquero et al., 2013; Carvalho et al., 2021). The plasmids also encoded various efflux pump proteins, such as *tet(E)*, merD, mexC, OprJ, mph(E), (Chopra, 2002), and mph(A) known to play significant roles in drug trafficking across cell membranes (Dayao et al., 2016; Kim et al., 2017; Yang et al., 2021). The presence of type II toxin-antitoxin RelEParE and T2SS is indicative that the plasmids also carry virulence genes. The presence of proteins such as the conjugal transfer protein TraF points to the presence of proteins that facilitate gene transfer between bacteria species. Other researchers (Majumdar et al., 2006; Preena et al., 2021) have noted that plasmids of aeromonads can be cured after sub-culturing, and depending on the history of the isolates after primary isolation, we may not have sequenced all plasmids of the original isolates in this study. Altogether, these observations show that *A*. *media* strains isolated from different geographical and host species in the world carry various multidrug efflux pump proteins, transposons, AMR, and virulence genes. However, this is need for *in vivo* studies using approaches such as mutagenesis, cloning, and purification of virulent genes identified in this study in order to determine their virulence mechanisms in different host species. Such studies would shed more insight on genes that are crucial for the pathogenicity of *A*. *media*.

## Conclusion

In this study, we have shown that *A*. *media* strain SD/21–15 isolated from marine sediments in Denmark shares several virulence genes such as adherence proteins, hemolysins, secretion system, iron acquisition, biofilm formation and quorum sensing genes with other *A*. *media* strains isolated from different host species and geographical areas in the world. We have also shown that strain SD/21–15 shares several genes like hemolysins, adherence proteins, and T2SS with other *Aeromonas* spp. although it lacks the cytotoxic *aerA* gene. The presence of *bla*_MOX-9_, *bla*_OXA-427_, *crp,* and *mcr* genes in all 25 isolates is indicative that these AMR genes are highly prevalent in *A*. *media* isolates found in different ecosystems. The presence of transposases, integrase, recombinases, virulence, and AMR genes in the plasmids is indicative that the *A*. *media* strains examined in this study had the potential to transmit virulence and AMR genes to other bacteria. In summary, our findings shed new insights on virulence genes and the role of *A*. *media* in the spread of AMR genes.

## Data availability statement

The datasets presented in this study can be found in online repositories. The link to the repository can be found below: https://www.ncbi.nlm.nih.gov/nuccore/JAJVCY000000000.1.

## Author contributions

SD, HS, and HM: conceptualization, methodology, supervision, data curation, bioinformatics analysis, and mobilizing resources. SD, EA-W, BP, ØE, HS, and HM: manuscript preparation, editing, and submission. All authors contributed to the article and approved the submitted version.

## Funding

This study was financed by the Research Council of Norway under the FIFOSA-21 Project Grant Number 320692.

## Conflict of interest

The authors declare that the research was conducted in the absence of any commercial or financial relationships that could be construed as a potential conflict of interest.

## Publisher’s note

All claims expressed in this article are solely those of the authors and do not necessarily represent those of their affiliated organizations, or those of the publisher, the editors and the reviewers. Any product that may be evaluated in this article, or claim that may be made by its manufacturer, is not guaranteed or endorsed by the publisher.

## Footnotes

^1^ https://usegalaxy.no/

^2^ https://usegalaxy.eu/

^3^ RStudio nmbu.no

^4^ https://proksee.ca/
